# How to improve your breast cancer program: Standardized reporting using the new American College of Radiology Breast Imaging-Reporting and Data System

**DOI:** 10.4103/0971-3026.57206

**Published:** 2009-11

**Authors:** Haydee Ojeda-Fournier, Judy Q Nguyen

**Affiliations:** Department of Radiology, Moores Cancer Center, University of California, San Diego La Jolla, CA, USA

**Keywords:** Breast, MRI, Ultrasound, Mammography

## Abstract

In the USA, the use of the American College of Radiology Breast Imaging-Reporting and Data System (ACR BI-RADS) has served not only as a quality assurance tool and guide to standardizing breast imaging reports but has also improved communication between referring physicians, researchers, and patients. In fact, in the USA, the Mammography Quality Standards Act of 1997 requires that all mammograms be assigned a BI-RADS category based on the finding of most concern. In this manuscript, we aim to review the recommendations provided in the 4^th^ edition of the ACR BI-RADS for mammography, USG, and MRI. We also review the major controversies surrounding the use of ACR BI-RADS.

## Introduction

The American College of Radiology (ACR) first developed the Breast Imaging-Reporting and Data System (BI-RADS) in 1993 in an effort to provide a quality assurance tool that would standardize mammographic reporting, facilitate outcome monitoring, and reduce the ambiguity surrounding breast imaging reports.[1,2] Before that, reports were not standardized and there was inconsistent use of imaging terminology, resulting in confusion to the referring physicians and patients. There was also no mandate to provide further patient management recommendations based on imaging findings. In many instances, reports could not be clearly categorized as either positive or negative. This created the additional challenge of the need to track outcomes and perform practice outcome audits. When the mammographic ACR BI-RADS was introduced, it was lauded for providing a standardized lexicon and reporting format. It allowed the radiologist to relate the degree of concern for malignancy through a concise description, using approved terminology, and to give clear management recommendations [Tables 1 and 2]. Since then, BI-RADS lexicons have been developed for USG and MRI and these were published in the 4^th^ edition of ACR BI-RADS.[2] It is now possible for not only researchers but also clinical practices to perform a full mammography audit by collecting the minimum raw data (i.e., number of examinations, recall numbers, biopsy results, etc.).[3–5] This has led to several studies demonstrating that the ACR BI-RADS assessment categories and lexicon have excellent correlation with the risk of breast malignancy.[1,6–8] Finally, the new ACR BI-RADS lexicon for breast MRI has been advocated for mainstream use as it facilitates clear and concise interpretation.[9]

**Table 1:** Summary of ACR BIRADS Approved Descriptors

	MAMMOGRAPHY
Masses	
Shape	Round, oval, lobular, irregular
Margins	Obscured, indistinct, spiculated, microlobulated, circumscribed
Density	High, isodense, low, radiolucent
Calcifications	
Benign	Usually large, round, coarse (popcorn-like), rod-like, lucent-centered, eggshell/rim, diffuse, scattered, bilateral, regional, dermal, vascular, milk of calcium, suture, dystrophic
Intermediate	Usually smaller, amorphous, indistinct, coarse heterogeneous, clustered, regional, linear, segmental
Suspicious	Punctate, fine pleomorphic, fine linear, fine-linear branching, segmental
Asymmetry	Global, focal
Special cases	Asymmetric tubular structure/solitary dilated duct, intramammary lymph node
Associated findings	
Used with masses, asymmetries, calcifications, or can stand alone as a finding	Skin or nipple retraction, skin thickening, trabecular thickening, skin lesion, axillary adenopathy, architectural distortion

	**ULTRASOUND**

Masses	
Shape and margins	Uses same terminology as in mammography where applicable
Orientation	Parallel, non-parallel, wider-than-tall, taller-than-wide
Lesion boundary	Abrupt interface, echogenic halo
Echogenicity	Anechoic, hyperechoic, hypoechoic, isoechoic, complex mixture
Posterior acoustic features	None, enhancement, shadowing, combined pattern
Effects on surrounding tissue	Compression, obliteration, straightening or thickening of Cooper's ligaments, edema, skin retraction/irregularity
Calcifications	Poorly characterized on US but can use descriptors similar to mammography
Macrocalcifications	>0.5mm, coarse, shadowing
Microcalcifications	Within the mass, outside the mass
Special cases	Clustered microcysts, complicated cysts, mass in or on skin, foreign body, intramammary or axillary lymph nodes
Vascularity	Present or not, increased, decreased, none

	**MRI**

Masses	
Shape	Round, oval, lobular, irregular
Margins	Smooth, irregular, spiculated
Internal enhancement	Homogeneous, heterogeneous, rim enhancement, dark internal septations, enhancing internal septation, central enhancement
Enhancements	
Focus/foci	<5mm
Non-mass-like	Focal, linear-non-specific, linear-ductal, branching-ductal, segmental, regional, diffuse, homogeneous, heterogeneous, stippled/punctate, clumped, ring-enhancing, reticular/dendritic, symmetric, asymmetric
Associated findings	Nipple retraction or inversion, pre-contrast high duct signal, skin retraction, skin thickening, skin invasion, edema, lymphadenopathy, pectoralis muscle invasion, chest wall invasion, hematoma/blood, abnormal signal void, cyst

**Table 2:** American College of Radiology BI-RADS Final Assessment Categories

BI-RADS categories	Assessment	Clinical management
0	Incomplete	additional mammographic views, comparison films, ultrasound, MRI are required
		once additional studies are completed, a final assessment can be formed
1	Negative	completely negative exam, no significant lesions, masses, architectural distortion, suspicious calcifications, etc
		normal-interval follow-up
2	Benign finding	normal assessment
		benign lesion present that carries no malignant potential and requires no intervention
		normal-interval follow-up
3	Probably benign finding	almost certainly benign lesion, carries <2% risk of malignancy
		biopsy not required
		short-interval follow-up (<1 year)
4	Suspicious abnormality	some form of intervention is required, either aspiration or biopsy
		4A – low suspicion for malignancy
		4B – Intermediate probability for malignancy, only truly benign if both radiologic and pathologic follow-up are benign
		4C – moderate concern for malignancy, but lesion is not classic for cancer, a malignant result is expected on biopsy
5	Highly suggestive of malignancy	almost certainly malignant, >95% probability of cancer
		classic characteristics for cancer
		percutaneous tissue sampling required for oncologic management
6	Known biopsy-proven malignancy	breast findings already proven by biopsy to be cancer but pending definitive treatment
		appropriate for patients seeking a second opinion, monitoring responses to neoadjuvant chemotherapy, or for patients who require further staging

## Report organization

The ACR BI-RADS includes recommended formats for various breast imaging reports, i.e., for mammography, USG, and MRI. This permits a concise and organized approach to image interpretation and reporting. Although there are differences between the imaging modalities with regard to their technical aspects, all reports are required to include a relevant clinical history, state the indication for the examination, list the pertinent findings, provide the overall impression, and assign a BI-RADS category. Pertinent findings are written in a succinct manner using standardized terminology from the latest ACR BI-RADS lexicon without embellishment. All findings are to begin with a comment on the overall composition of the breast, followed by descriptions of any lesions that may be present. Features unique to a particular study, such as calcifications with mammograms or echogenicity with USG, are then described using approved descriptors as per the BI-RADS lexicon. The final impression needs to clearly state the degree of suspicion for malignancy and offer recommendations for further management based on the BI-RADS classification. Although more than one pertinent feature may have been observed, the final BI-RADS assessment is based on the most suspicious feature.

## Mammographic report

A standard mammogram report includes:

1. Clinical history
2. Indication for examination
3. Comparison with previous studies (if deemed necessary by the radiologist)
4. Breast composition
5. Finding(s)
6. Overall assessment and management recommendations

Indication for the examination could be either ‘screening’ or ‘diagnostic evaluation.’ A screening mammographic examination is performed on an asymptomatic woman in order to detect early, clinically unsuspected, breast cancer. Two standard views of each breast – mediolateral oblique and craniocaudal projection – are obtained. A diagnostic mammographic examination is performed on a woman who presents with clinical signs or symptoms. A diagnostic study is tailored by the radiologist to address the presenting complaint and may include additional projections other than the standard views; USG or, in rare cases, breast MRI may also be performed.

**Figure 1:**
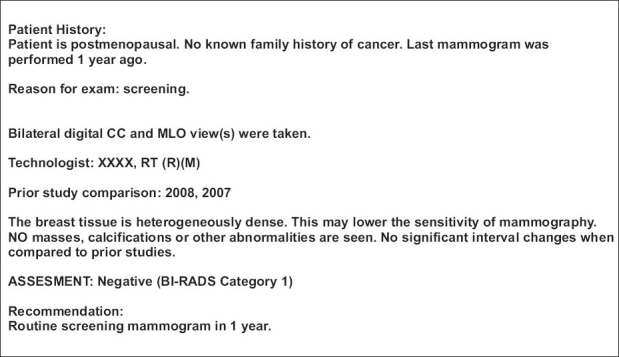
Example of a normal mammogram report in a patient with heterogenous breast density

The report on the mammographic findings begins with a description of the breast composition, commenting on background glandularity: fatty, scattered fibroglandular densities, heterogeneously dense, or extremely dense tissue. When the breast tissue is either heterogeneously dense or extremely dense, mammography has relatively low accuracy and a disclaimer statement can be added to the report regarding the decreased sensitivity of the study. BI-RADS-approved terminology is clearly defined in detail below. In general, descriptions of masses, calcifications, densities, and associated findings include details of size, morphology, and location. An example of a standard normal screening mammogram report in a heterogeneously dense breast is presented in Figure 1.

## USG report

A USG report is organized similar to a mammography report and includes:

1. Clinical history
2. Scope of examination
3. Comparison with previous studies (if available and pertinent)
4. Background echotexture
5. Finding(s)
6. Correlation with physical findings, mammography, MRI, etc.
7. Overall assessment and management recommendations

USG analysis begins with a brief examination of breast tissue in the area of concern. Any lesion identified is described in terms of size in at least two dimensions (in three dimensions, if the volume of the mass is analyzed in successive studies), a consistent and reproducible assessment of lesion location (o'clock position and distance from the nipple), and characterization of the lesion using ACR-approved descriptors. There should also be a statement correlating USG results with physical findings or other imaging findings if relevant.

## Breast MRI report

The breast MRI report includes:

1. Clinical history
2. Comparison with old studies
3. Description of the MRI technique used, including post-processing technique
4. Finding(s)
5. Kinetic curve report
6. Overall assessment and management recommendations

Description of the MRI technique includes relevant factors such as the use of a dedicated breast coil, pulse sequences, contrast dose, etc. In the ‘findings’ section, an overall assessment of the glandular tissues is made, similar to the assessment of background density in mammography or assessment of echotexture in USG studies. Terminology used includes: mild, moderate, or marked background glandularity. These statements can then clarify any questions regarding a lesion being hidden by normal tissue on other studies. Any artifacts that can affect interpretation can be described. Breast implants are described to include composition (saline or silicone) and number of lumens. Unique findings on MRI include enhancement, which is further characterized as focus/foci, mass, non-mass-like enhancement, and/or symmetric/asymmetric. The final impression should fully assess each lesion as a three-dimensional structure along with its kinetic findings. If an abnormality is deemed suspicious, the report indicates whether a biopsy should be considered and specifies the recommended image guidance for the procedure (i.e., stereotactic, USG, or MRI).

If more than one type of breast imaging modality is being interpreted, a single report that integrates all significant findings is recommended in order to better guide clinical management.

An integrated report would include the following:

1. Clinical history
2. Comparison with previous studies (if available and pertinent)
3. Statement of scope of examination (targeted or survey) and technique used
4. Type and order of the different studies comprising the overall examination (include brief statement of rationale for each)
5. Correlation with physical, mammographic, or MRI findings
6. Overall assessment
7. Management recommendations

Sometimes, it is necessary to split findings for two or more imaging modalities into separate reports for coding and reimbursement reasons, but it is recommended that the final assessment integrate findings from all current imaging studies.

## Lexicon

The ACR Committee on Breast Cancer has addressed the widespread confusion caused by evolving terminology, which was neither universal nor clearly defined and, oftentimes, was used redundantly. An approved standardized lexicon [Table 1] was reviewed and adopted into the ACR guidelines with the hope of decreasing confusion and promoting universal utilization among breast imagers. By using the ACR-approved descriptors, breast imaging reports have become more streamlined and clear and concise. Both USG and MRI have unique features not found in mammography but, wherever applicable, common features are described using the terms developed for mammography. BI-RADS assessment categories however are the same for all three imaging modalities.

## Mammogram

**Figure 2(A-C):**
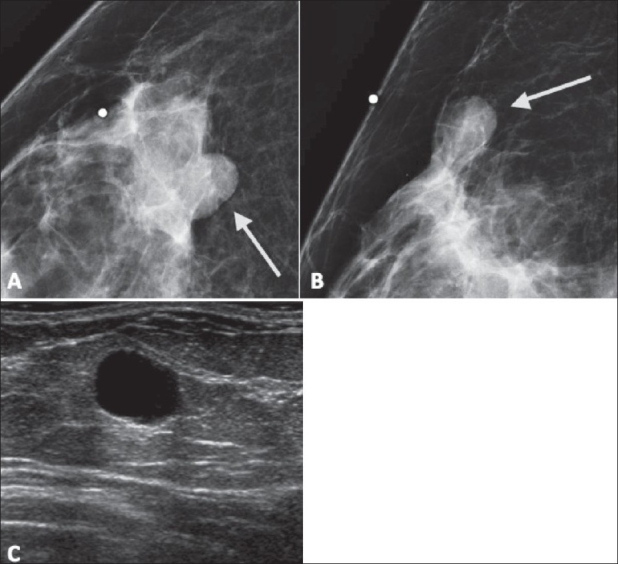
A 37-year-old female with a palpable breast mass marked with a BB at the time of diagnostic evaluation. Mammogram (A,B) demonstrates a partly obscured, partly circumscribed, oval mass (arrow). Follow-up USG (C) demonstrates an anechoic structure with imperceptible margins and posterior enhancement consistent with a simple cyst. This was classified as BI-RADS 2

By definition, a mass is a space-occupying lesion seen in two different projections and, further, is characterized by shape, margin, and density [Figure 2].

**Figure 3:**
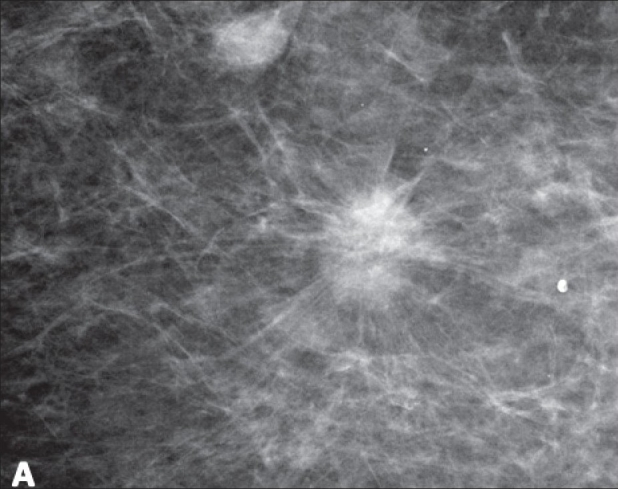
Screening mammography in a 67-year-old woman demonstrates a spiculated mass. Biopsy showed invasive ductal carcinoma, nuclear grade II. This was classified as BI-RADS 5
- Density descriptors include terms like high, equal, low, or fat-containing and are used to describe X-ray attenuation of the lesion relative to the expected attenuation of the surrounding fibroglandular breast tissue. For example, Figure 4 demonstrates a fat-containing lesion.

- The shape of a mass is classified as round, oval, lobular, or irregular.
- Margins can be obscured, indistinct (ill-defined), spiculated (as demonstrated in Figure 3), microlobulated, or circumscribed (well-defined or sharply-defined). Incidentally, the descriptor ‘circumscribed’ can be used as long as 75% of the margin is clearly visible, with the remainder being no worse than obscured by the overlying tissue.

**Figure 4:**
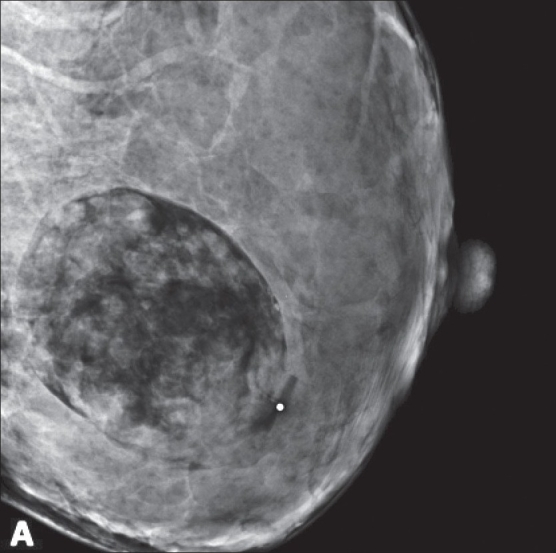
A 26-year-old pregnant female with a new palpable mass. A single mediolateral oblique projection was obtained, which demonstrates a fat-containing oval mass, pathognomonic of hamartoma, classified as BI-RADS 2
- Asymmetry refers to a potential mass that is seen in only a single projection and is planar, lacks convex borders, and usually contains interspersed fat. Asymmetries lack the conspicuity of a three-dimensional mass. The term density has also been used for this same finding but is discouraged by the ACR as it has led to confusion because the term is also used to describe a mass' attenuation characteristics. A review of previous films is critical for accurate interpretation of asymmetries and, if none are available, a full diagnostic evaluation is warranted.
- ‘Global asymmetry’ involves a large portion of the breast (at least a quadrant) and is judged in comparison with a corresponding area in the contralateral breast. There is no mass, distorted architecture, or associated suspicious calcifications.
- ‘Focal asymmetry’ involves a smaller portion of the breast than does global asymmetry and does not fit the criteria of a mass [Figure 5]. It is a confined asymmetry, with a similar shape on two views, and it lacks the borders and the conspicuity of a true mass.

**Figure 5:**
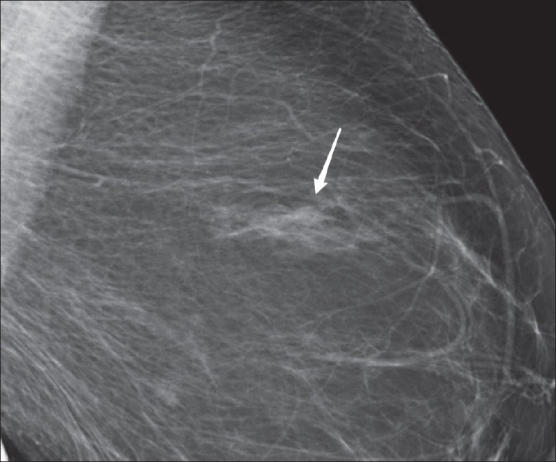
A 61-year-old woman with a stable asymmetry (arrow) in the upper outer quadrant of the left breast
- Calcifications need to be described by their size, morphology, and distribution.
- Benign morphology descriptors include amorphous, dermal, vascular, popcorn-like, rod-like, round (>1 mm), punctate (<0.5 mm), lucent center, eggshell/rim, and milk of calcium (sedimented calcifications in macro or microcysts).
- Amorphous calcifications are small and hazy and warrant further investigation with biopsy. Suspicious calcifications are described as fine linear, linear branching, and pleomorphic [Figure 6].

**Figure 6:**
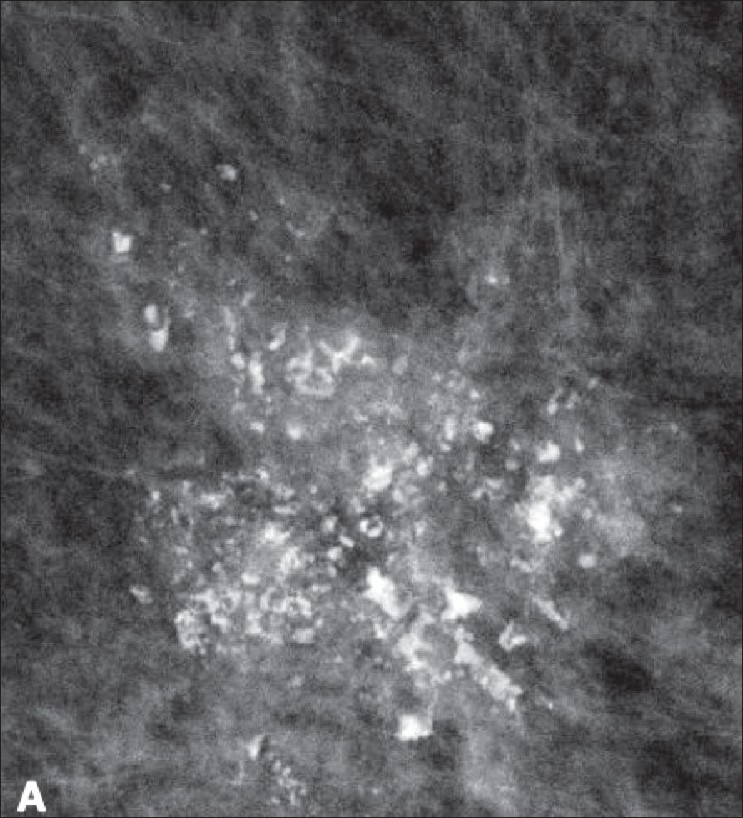
Screening mammogram in a 63-year-old woman shows clustered, pleomorphic microcalcifications, classified as BI-RADS 5. Biopsy showed high-grade ductal carcinoma *in situ* (DCIS)
- Descriptors for distribution patterns include grouped or clustered, segmental, regional, or diffuse/scattered. ‘Grouped/clustered’ should be used when at least five calcifications occupy a small volume (<1 cc) of tissue.
- Suture calcifications are uncommon now because suture material used currently causes less reaction than the catgut suture material used in the past, which would often develop deposits of calcium, making the knots visible.
- Dystrophic calcifications are common in the post-irradiated or post-trauma breast [Figure 7]. They are usually irregular in shape, >0.5 mm in size, and often have lucent centers.

**Figure 7:**
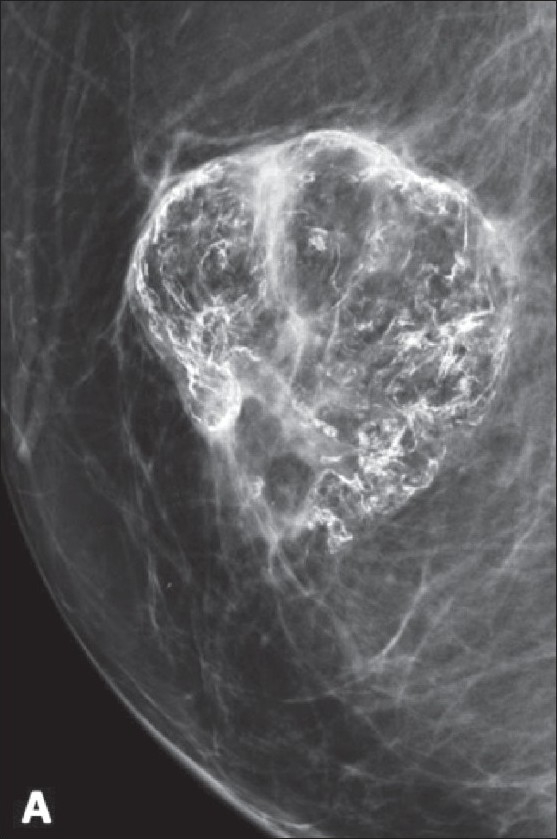
A 52-year-old woman presented with a palpable abnormality following breast reduction surgery. Mammography shows coarse and eggshell calcification consistent with fat necrosis, classified as BI-RADS 2

**Figure 8(A,B):**
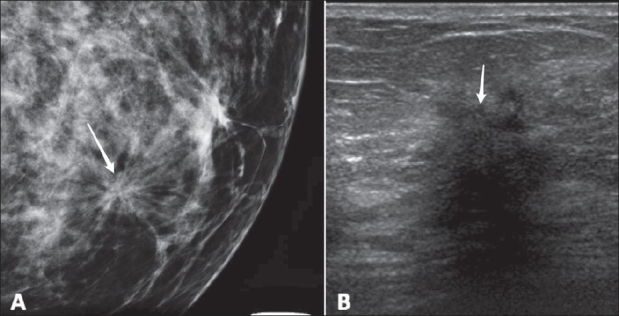
Mammography (A) in a 65-year-old woman shows architectural distortion. USG (B) demonstrates a hypoechoic mass with spiculated margins and posterior shadowing (arrow), classified as BI-RADS 5. Pathology showed invasive ductal carcinoma

Architectural distortion is considered an associated finding and the term is used when the normal breast tissue architecture is distorted but there is no definite mass. It is visualized in the form of spiculations radiating from a point or as focal retraction or distortion of the edges of the parenchyma [Figure 8].

Associated findings may occur in conjunction with masses, asymmetries, or calcifications or may be stand-alone findings. Such findings include skin/nipple retractions, skin thickening (either diffuse or focal), trabecular thickening, skin lesions, and axillary lymphadenopathy.

## USG

Given the success of the standardized mammogram lexicon, the ACR developed a similar lexicon for USG in 2003. Research has shown the success of the USG BI-RADS lexicon in improving communication, diminishing confusion, and facilitating reporting and data tracking.[5]

Background echotexture is unique to USG and is characterized as heterogeneous or homogeneous. Homogeneous echotextures can be further described as fibroglandular or fat.

The shape and margins of masses are described using the same descriptors as are used for masses in mammography. Pertinent descriptions of masses should also include distinctive USG features, such as:

- Posterior acoustic qualities, which may be described as “none,” “enhancement,” or “shadowing” (as seen in Figure 8B), or may be a combination of these
- Orientation (parallel or not parallel to the skin edge)
- Echo pattern [hyperechoic, anechoic (as seen in Figure 2C), hypoechoic, isoechoic, or a complex combination of these]
- Lesion boundary describes the transition zone between the mass and the surrounding tissue and may be an abrupt interface or an echogenic halo
- Effects of the mass on surrounding tissue (compression, obliteration, effects on Cooper ligaments, echogenic halo, edema, etc.)
- USG is not ideal for characterizing calcifications; they are seen as echogenic foci, particularly when within a mass.
- There are several special cases described by the USG lexicon:
- Clustered microcysts consist of tiny anechoic foci, individually smaller than 2–3 mm, with thin (<0.5 mm) intervening septae and no discrete solid component.
- Complicated cysts may contain brightly echogenic foci that scintillate as they shift through fluid-debris levels. These cysts do not contain solid mural nodules. If a discrete solid component is identified, the lesion should be classified as a complex mass requiring aspiration or other intervention.
- Masses in or on the skin, such as epidermal inclusion cysts, keloids, moles, etc., can also be identified.
- Foreign bodies include marker clips, coils, wires, catheter sleeves, silicone (with a characteristic ‘snowstorm’ appearance), and metal or glass related to trauma.
- Intramammary and axillary lymph nodes.
- Vascularity should be described in terms of whether it is present or not, location in relation to lesion, and extent.

## MRI

The MRI lexicon has evolved over the last 4 years and remains work-in-progress. The current ACR BI-RADS MRI lexicon reflects current technology and will be periodically updated as new sequences and imaging techniques develop. The ACR has emphasized that breast MRI images are representations of three-dimensional objects and that multiplanar reconstructions and three-dimensional images are appropriate for review; therefore, use of a software package for breast MRI interpretation is recommended. Kinetic information is as important as morphologic descriptions of significant lesions and the availability of a software package to interpret breast MRI aids in the display of color overlay and curve assessment.

**Figure 9(A-C):**
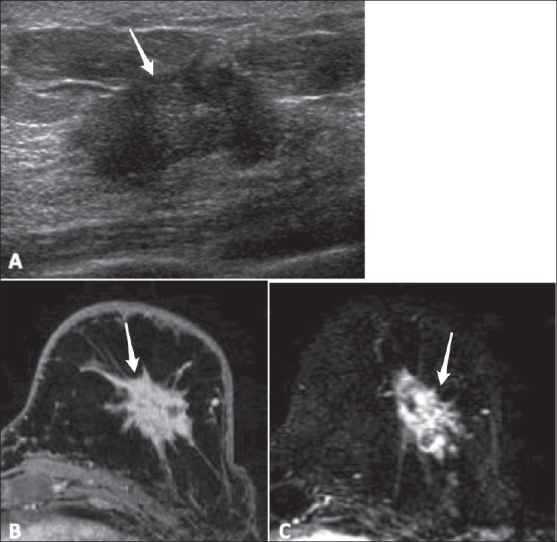
A 55-year-old woman with a palpable abnormality. USG (A) shows a hypoechoic, irregular mass (arrow) with angular margins, classified as BI-RADS 5. Biopsy of the mass showed stage II invasive ductal carcinoma. A pre-operative breast MRI with high-resolution delayed post-contrast (B) and subtracted (C) images, demonstrates a heterogeneously enhancing, irregular mass (arrows) with spiculated margins, classified as BI-RADS 6

The terms ‘mass’ and ‘regional enhancements’ have been a source of confusion. A mass has well-defined margins, with distinct edges separating it from the surrounding glandular tissue. This is demonstrated on USG and MRI in Figure 9. It is usually related to a pathological process in a ball-like, three-dimensional structure that may or may not displace or otherwise affect normal breast tissue. Masses can be further described using characteristics common to mammography and USG, such as margins, basic geometry, size, location, and associated findings, in addition to features unique to MRI, such as enhancement pattern and kinetics.

**Figure 10:**
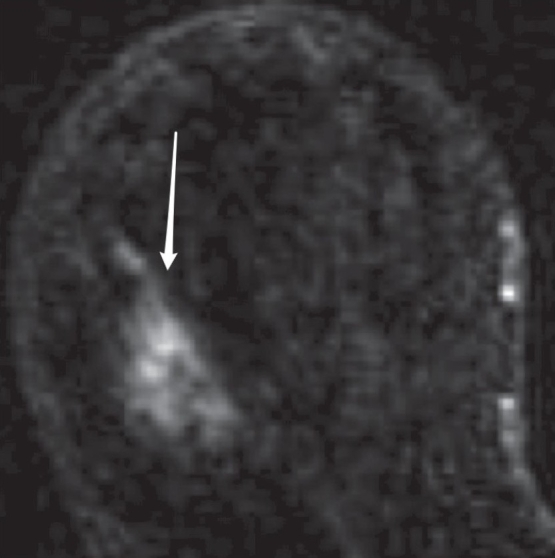
A 61-year-old woman with a history of ductal carcinoma *in situ*. Breast MRI demonstrates an area of non-mass-like enhancement (arrow), classified as BI-RADS 4. MRI-guided biopsy revealed benign fibrocystic changes
- The internal characteristics of non-mass-like enhancements are further described as homogeneous, heterogeneous, stippled/punctate, clumped, or reticular/dendritic. Stippled refers to multiple, often innumerable, punctate foci that are approximately 1–2 mm in size and appear scattered throughout an area of the breast. It does not conform to a duct system. The clumped pattern refers to an aggregate of enhancing masses or foci in a cobblestone pattern that may occasionally be confluent. Reticular/dendritic enhancement is a spider web-like pattern found among women who have undergone at least partial involution of the glandular tissue, leaving strands of breast tissue among strands of fat. It appears as bright thickening, distortion, and foreshortening of normal fibroglandular trabeculae and tissue, with loss of the normal scalloped edges of the fat/breast tissue interface at its edges
- ‘Symmetric’ and ‘asymmetric’ are used when bilateral breast MRI is performed. Symmetric refers to mirror-image enhancement in the right and left breasts. Asymmetric enhancement [Figure 11] is a more concerning finding and should be further modified using the lexicon described above.

- Shapes can be round, oval, lobulated, or irregular
- Margin analysis is dependent on spatial resolution and can be described as smooth, spiculated, or irregular (it is strongly recommended that the descriptor ‘irregular’ be used to characterize either the margin or the shape, but not both, to avoid confusion). Figure 9 shows a heterogeneously enhancing mass with an irregular shape and spiculated margins
- As with other breast imaging modalities, size and location should be reported in a consistent, reproducible manner
- Enhancement is defined as higher signal intensity compared with the surrounding normal glandular tissue after contrast administration. The size and location should be reported along with any associated findings
- If a mass exhibits internal enhancement, it is described as homogeneous or heterogeneous. Homogeneous enhancement is confluent and uniform. Heterogeneous enhancement is non-uniform, with areas of variable signal intensity. Additional descriptors of patterns of enhancement of a mass are rim enhancement, dark internal septae, enhancing septae, or central enhancement.
- A ‘focus,’ on the other hand, is a tiny punctate enhancement that is non-specific and too small (<5 mm) for morphological characterization. There is usually no corresponding finding on the pre-contrast scan. Foci can be multiple spots or dots of enhancement, separated widely in the breast by normal tissue or fat, and are not a conglomerate of dots in one small area.
- An area of non-mass-like enhancement is neither a focus nor a mass and its pattern can extend over small or large regions in a pattern that can be focal, linear, ductal, segmental, regional, multiple regions, or diffuse. A focal area of abnormal enhancement is <25% of a breast quadrant volume and comprises a single abnormal enhancement pattern. Ductal enhancement can be linear or branching and corresponds to one or more ducts, usually radiating toward the nipple. Segmental enhancement refers to a triangular or cone-like shape with the apex at the nipple. Regional enhancement may be geographic and lacks convex borders, as demonstrated in Figure 10. To qualify as multiple regions of enhancement, there must be at least two broad areas separated by normal tissue or fat. Lastly, diffuse non-mass-like enhancement is widely scattered and evenly distributed

**Figure 11:**
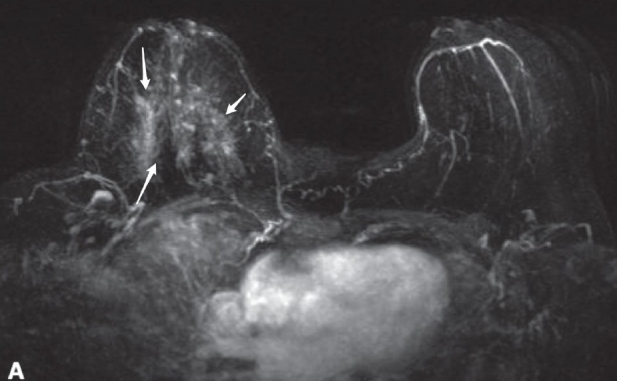
Pre-operative breast MRI in a 62-year-old woman with biopsy-proven high-grade ductal carcinoma *in situ* demonstrates diffuse non-mass-like enhancement (arrows) in the right breast, classified as BI-RADS 6
- Kinetic techniques analyze dynamic lesion enhancement and generate signal intensity/time curves. The shapes of these curves offer a wealth of information to help differentiate between benign and malignant lesions
- The initial enhancement phase is analyzed within the first 1–2 min after injection. The descriptors are slow, medium, or rapid
- The delayed phase refers to the enhancement pattern after 2 min or after the curve starts to change. This curve is described as persistent, washout, or plateau. Persistent refers to continued increase in signal intensity over time. Washout describes a curve that shows decreasing signal intensity after peak enhancement. A plateau curve reaches the maximum signal intensity and then remains constant

**Figure 12(A-E):**
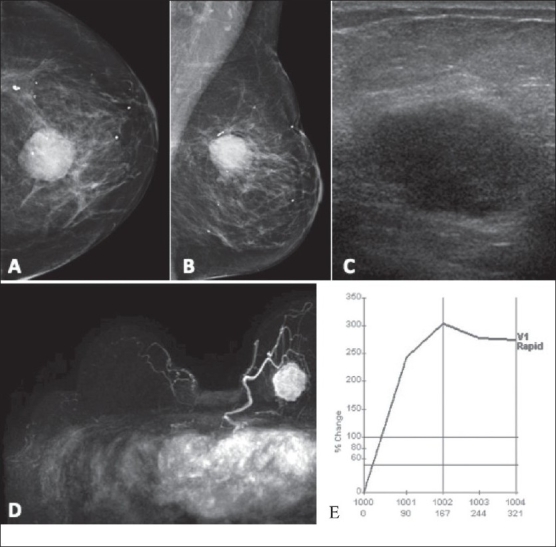
A 66-year-old woman with a remote history of bilateral breast cancer and radiation therapy presented with a new rapidly growing mass. Craniocaudal (A) and mediolateral oblique (B) mammograms show a round mass. USG (C) demonstrates a hypoechoic oval mass with no posterior enhancement, classified as BI-RADS 5. Biopsy showed sarcoma. Pre-operative MRI (D) shows a round mass with a lobulated margin, with no chest wall invasion. Kinetic assessment (E) demonstrates rapid initial enhancement followed by washout in the delayed portion of the curve, classified as BI-RADS 6

In Figure 12, a biopsy-proven malignant mass is pre-operatively evaluated with MRI, including kinetic assessment. There is a rapid initial enhancement, followed by a washout signal intensity curve.

## Overall assessment and recommendations

The key component of the breast-imaging report is the overall assessment and recommendations [Table 2]. A single BI-RADS category is assigned based on the most suspicious finding on imaging. If more than one imaging study is performed, the final assessment incorporates findings from all these studies.

There are seven assessment categories: ACR BI-RADS categories 0 through 6. These assessment categories are divided into incomplete (category 0) and final (categories 1–6).

### Category 0: Assessment is incomplete

These examinations are incomplete until some further evaluation is performed. This can be in the form of additional mammographic views, comparison films, USG, or, less commonly, breast MRI. At times, in order to accurately assign a final BI-RADS classification, comparison with old films is required. The radiologist should use judgment on how vigorously to attempt to find old films for comparison and whether the report truly needs a category 0 coding *vs* category 1 or 2. Tracking old films can be time consuming and expensive and therefore ‘category 0 (for comparison)’ should only be used when such comparison is absolutely required for making a final assessment. Once comparisons or additional imaging studies are completed, a final assessment can then be rendered. Category 0 is often used in a screening situation.

### Category 1: Negative

This is a completely negative exam with no significant findings. There are no masses, no architectural distortion, no suspicious calcifications, and no asymmetries.

### Category 2: Benign

This is a normal assessment in which the radiologist describes a benign lesion that carries no malignant potential. Examples include cysts [Figure 2], lipomas, dystrophic calcifications [Figure 7], hamartomas [Figure 4], intramammary lymph nodes, implants, and many other benign findings of no clinical consequence.

### Category 3: Probably benign

**Figure 13:**
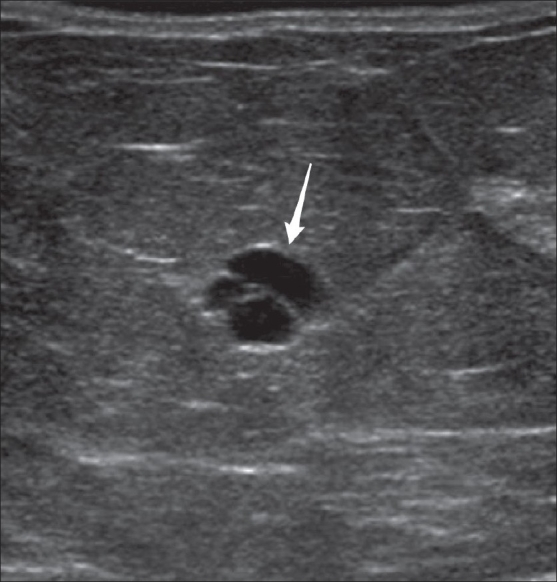
Incidental, non-palpable complicated cyst on USG, classified as BI-RADS 3. A short interval, 6-month follow-up is requested to assess stability

Category 3 remains a source of confusion and is sometimes controversial. This category is to be used in the presence of a finding that is almost certainly benign but for which a short follow-up is desired. It is never to be used as an indeterminate malignancy category or in lieu of a diagnostic work-up. In fact, this category has a <2% risk of malignancy and is unlikely to require biopsy. The finding is not expected to change quickly over time and therefore recommended follow-up involves a series of short-interval (6-month) follow-ups over a period of 24–36 months. After the finding has maintained a stable appearance for 2–3 years, it can be considered benign and be coded as a category 2 (benign) finding. In some instances, due to patient and/or clinician concern, some category 3 findings may end up being biopsied rather than followed. This category is not to be used when there is a palpable lesion. Examples of category 3 lesions include non-palpable, incidental, complicated cysts [Figure 13].

### Category 4: Suspicious abnormality

This category includes findings that do not have a classic appearance for malignancy, but have a higher probability for malignancy than findings classified as category 3. Here, some form of intervention (preferably image-guided needle core biopsy) is recommended to establish a diagnosis. The wide range of outcome probabilities has stimulated a subdivision of category 4. An optional form of subdivision suggested by the ACR is 4A, 4B, and 4C. A 4A coding can be used for a finding with a low suspicion for malignancy but requiring some type of intervention. Examples include a palpable complicated cyst or probable abscess. Category 4B includes lesions with intermediate probability for malignancy. Such a lesion requires close radiologic and pathologic follow-up and is only truly benign if the results of both concur. Examples include fat necrosis and papilloma. Category 4C identifies findings that are of moderate concern but do not exhibit the classic signs of malignancy. A malignant result is expected in this category and it should alert pathologists and clinicians to carefully follow-up on these biopsies. Included here are ill-defined, irregular solid masses or new clusters of fine pleomorphic calcifications.

### Category 5: Highly suggestive of malignancy

These lesions are almost certainly malignant, carrying a >95% probability of malignancy. Imaging findings exhibit the classic characteristics of malignancy and percutaneous tissue sampling may be required for oncologic management (i.e., neoadjuvant chemotherapy) or to plan a one-stage definitive surgical intervention that may include lymph node sampling. A spiculated, irregular, high-density mass would be a classic example of category 5 [Figure 3].

### Category 6: Known biopsy-proven malignancy

This category is new and was added to accommodate breast findings that have been proven to be cancer by biopsy but for which definitive treatment (surgical excision, radiation, chemotherapy, or mastectomy) has not yet been executed. It is appropriate for patients seeking a second opinion, for monitoring responses to neoadjuvant chemotherapy, or for patients who require further staging.

The final assessment is always based on the most immediate action required. For instance, a patient with known cancer in one breast may be sent for an outside imaging consultation. In the event that there are no other significant findings apart from the cancer or if there is a benign finding that requires no intervention, the report can be classified as category 6. If another abnormality is found that requires further evaluation, the final assessment would then be category 0. If there is an additional lesion requiring biopsy, then the report would be coded category 4.

In terms of the mammography audit, BI-RADS have helped to more clearly define positive and negative studies. Any screening mammogram coded BI-RADS categories 0, 4, or 5 is considered positive. Categories 1, 2, and 3 are negative. In the scenario of a patient with negative imaging but a clinically significant palpable finding, the report should still be coded based on the imaging findings alone. However, the final assessment should take into consideration the clinical findings and make appropriate recommendations. All breast imaging centers in the USA are required by the Mammography Quality Standards Act (MQSA) to perform a basic medical audit to track sensitivity, specificity, positive and negative predictive values, cancer detection rate, and abnormal interpretation rates. A chapter in the 4^th^ edition of the ACR BI-RADS manual provides guidance for follow-up and outcome monitoring.

## Controversies

Multiple studies have shown that the ACR BI-RADS lexicon is effective and that the positive predictive value for each of the BI-RADS assessment categories is for the most part consistent among different radiologists.[7,8,10] In the beginning, several weaknesses were noted, including confusion regarding the system's guidelines. This was partly attributed to the fact that the guidelines do not explicitly state how to make a final assessment based on mammographic features. Also, the approved terminology was initially thought to be restrictive or unsuitable.[8] Despite initial drawbacks, BI-RADS has been an excellent system and, for the most part, has accomplished its goal of clearing up ambiguity surrounding breast imaging reporting. Acceptance, since its introduction in 1993, has universally increased. Since the 1997 MQSA was passed, assignment of the ACR BI-RADS category based on the finding of most concern is now required in the USA for all mammograms.[1] Since 1993, ACR BI-RADS has undergone several revisions, including the addition of USG and MRI lexicons and refinement of the lexicons and assessment categories to better accommodate clinical needs.

Revisions have been guided in part by studies demonstrating intra- and interobserver variability. It has been consistently noted that intraobserver agreement is better than interobserver agreement.[10] The reason seems to be that radiologists have their own personal interpretations of BI-RADS, varying thresholds, and different cut-off points in determining the best-fit descriptors and categories.[10] Taplin, Lehman, and others have shown that interobserver variability is not significant in the negative and benign assessment categories but is statistically significant in assessments and management recommendations associated with BI-RADS category 3 and, to a lesser extent, categories 4 and 5.[3,8,11,12] Although lesion description may vary from observer to observer depending on personal preferences and experience, it is critical that the final assessment category result in the correct action.

With regard to BIRADS category 3, the ‘probably benign’ finding, there is high variability in its usage, in both academic and community practices. This category is reserved for findings where the risk of malignancy is low (<2%) and short-interval follow-up is preferred over biopsy in almost all cases. Lehman and colleagues, however, demonstrated that category 3 lesions in a community practice had an actual cancer detection rate of 8.8%.[6] Upon further investigation, the criterion for a probably benign finding was not strictly adhered to in at least 80% of the examinations. The factors cited for this misuse included misclassification of morphologic features, failure to take into account available prior images, or failure to properly track significant changes in the lesion over time.[6] Taplin observed similar improper usage of category 3-coded examinations, where 37% had recommendations for ‘additional imaging’ and another 19% were referred for ‘normal-interval follow-up.’[12] In Taplin's study, improper usage was also seen in category 4 (38%) and category 5 (7%), for which additional imaging was recommended instead of the appropriate recommendation of a biopsy. To summarize, although category 3 remains controversial, it is to be used only after complete problem-solving imaging has been performed and only then is the lesion in question deemed probably benign.

Category 0 also has limitations as it covers a broad range of results, ranging from those needing comparisons to assess stability to those recommending further evaluation of benign or highly suspicious lesions. Taplin suggests a modified category 0 to better describe the level of concern.[12] Modifiers such as ‘incomplete assessment of a probably benign finding,’ where imaging would be performed to clarify an ambiguity and ‘incomplete assessment of a suspicious finding,’ where additional imaging would lead to a category 4 or 5 coding, may be useful in the future.

In the 4^th^ edition of the ACR BI-RADS, a subcategorization of category 4 lesions is recommended by the ACR, although it is not explicitly required. Before this, BI-RADS category 4 was a heterogeneous collection of lesions with a wide range of malignancy possibilities and thus could not clearly describe the radiologist's degree of concern.[13] By dividing category 4 into 4a (low), 4b (moderate), and 4c (high), the pre-biopsy risk of malignancy is better communicated to the referring physician, pathologist, and radiologist who may be involved in performing the follow-up biopsy. Berg found that it is the lesions near the threshold for biopsy that are the most problematic and were probably responsible for the high variability in actual practice.[14] Subdivision of category 4 is expected to address some of this variability.

Berg has found that specialized BI-RADS training improves the interobserver agreement among breast imagers with respect to lesion description and the final assessment.[3] Other researchers agree with Berg's findings and also feel that periodic performance assessments and self-auditing practice tests will aid consistent use of the ACR BI-RADS system.[10,12,14]

Implicit in the ACR BI-RADS system is the fact that the lexicon and assessment categories are to be consistently used among radiologists, even if it requires additional training to become familiar with the system. For example, the MRI and USG lexicons are new additions to BI-RADS that still need optimization and consistent use among radiologists.[5,15] This is recognized by the ACR and it is clearly stated in their guidelines that the BI-RADS lexicon remains a ‘living document’ that will certainly undergo future revision. As new imaging modalities emerge, the BI-RADS lexicon will also evolve.

## Conclusion

By consistently and appropriately utilizing the standard lexicon and assigning the appropriate ACR BI-RADS final assessment categories, radiologists specializing in breast imaging will effectively communicate findings, degree of concern for malignancy, and recommendations to both clinicians and patients. Although problems persist with the system, including the issues of inter- and intraobserver variabilities, multiple studies have validated the efficacy of the descriptors and assessment categories. There is no other system available currently that accomplishes what BI-RADS has done. A return to the situation prior to BI-RADS, when ambiguous reports were common, is not desired by anyone. Continued auditing, research, and refinement and revisions of the BI-RADS lexicon are to be expected and will lead to continued improvement and better patient care.
